# Case report: Ocular malformation with a ‘double globe’ appearance

**DOI:** 10.4103/0971-3026.57213

**Published:** 2009-11

**Authors:** Usha R Kim, Vipul Arora, Akash D Shah, KG Srinivasan

**Affiliations:** Aravind Eye Hospital, Madurai, Tamil Nadu, India; 1 K.G.S Scans, Anna Nagar, Madurai, Tamil Nadu, India

**Keywords:** Colobomatous cyst, coloboma, double globe

## Abstract

Colobomatous cyst of the orbit is a rare congenital cystic malformation associated with ocular maldevelopment. Usually, the cyst is associated with a microphthalmic globe. We present a rare case of a unilateral large colobomatous cyst associated with a normal-sized globe, giving the appearance of a double globe on imaging.

## Introduction

A colobomatous cyst is defined as a neuroectoderm-lined mass that protrudes through a coloboma in the wall of a microphthalmic eye.[1] It is usually diagnosed at birth and can affect one or both globes.[2] We present a rare case of a 2-year-old male who had bilateral corneal opacities and a right-sided colobomatous cyst with a normal-sized globe.

## Case report

**Figure 1:**
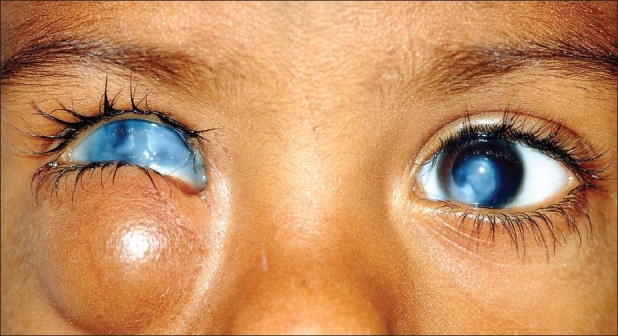
Photograph of the patient on presentation. There is a globular swelling under the right lower lid that has pushed the globe upward. The patient also has bilateral corneal opacities

A 2-year-old child presented with a swelling under the right lower lid and corneal opacities in both eyes since birth [Figure 1]. The swelling was round and displaced the globe outwards and upwards. The child also had a history of cleft lip and cleft palate, which had been repaired at the age of 6 months. There was a history of delayed milestones and mild mental retardation. There was no history of consanguineous marriage between the parents.

**Figure 2:**
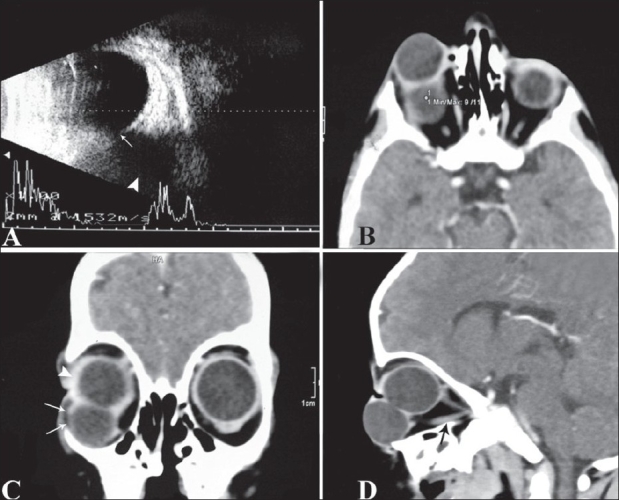
(A-D): B-mode USG (A) shows a well-defined cystic lesion (arrowhead) located in the inferior quadrant of the orbit with posterior acoustic enhancement, communicating with the globe (white arrow). Axial (B), coronal (C) and oblique sagittal multiplanar reconstruction (D) CT scan images show a colobomatous cyst on the right (arrows) with a normal-sized globe (arrowheads), the inferior rectus (black arrow in D) mimicking an optic nerve and giving rise to the appearance of a ‘double globe’

An external ocular examination of the right eye showed a formed globe with a corneal opacity. The left eye had a central and inferomedial corneal opacity with a normal anterior segment [Figure 1]. B-scan USG of the right eye was suggestive of a cystic lesion in the inferior quadrant, communicating with the eyeball [Figure 2A]. On A-scan, the axial length of the right eye was 22 mm and that of the left eye was 23 mm. A CT scan showed a coloboma of the posterior coat and the optic nerve head of the right eye. There was a large cystic lesion situated in the inferior compartment communicating with the globe. Together, these lesions constitute colobomatous cystic eye disease. The cyst wall was uniformly thin, with a CT density equal to that of normal sclera. The cyst cavity was homogenous, with no enhancement. The globe size was 2.31 × 2.11 cm and the size of the cyst was 2.50 × 2.37 cm [Figures 2B–D]. There was thinning of the optic nerve of the right eye. No obvious pathology was noticed in the left orbit. Oblique sagittal multiplanar reconstruction [Figure 2D] showed the inferior rectus muscle extending from the cyst to the apex of the orbit, thus mimicking the optic nerve. Because both the eye and the cyst were almost of the same size, this gave the appearance of a ‘double globe’ on the right side [Figure 2D]. There was mild dilatation and splaying of the lateral ventricles, with thinning of the corpus callosum.

The patient was diagnosed as a case of bilateral corneal opacities with a colobomatous cyst communicating with the globe in the right eye. He underwent an inferior transconjunctival orbitotomy, with aspiration of the contents of the cyst to reduce its volume, followed by excision of the cyst and suturing of the defect in the globe.

Histopathology of the cyst revealed the outer wall consisting of dense fibrocollagenous tissue. The cyst wall was lined with round to oval cells of neuroectodermal origin.

## Discussion

A majority of the cases of colobomatous cyst are associated with microphthalmos. There is no predilection for any particular sex. The condition is usually unilateral.[2] The etiology of a colobomatous cyst is not exactly known but it is presumed to occur due to improper fusion of the embryonic fissure between the 7–14 mm stage of fetal development. This results in abnormal ectasia of the sclera, which expands into the adjacent orbit.[3] Usually, the uveal contents do not develop in the region of the coloboma.

Shields and associates reviewed 645 orbital biopsies and reported that microphthalmos and colobomatous cyst accounted for 2% of 193 orbital cystic lesions and <1% of 645 biopsies.[4] They classified congenital cystic lesions of the orbit into various subgroups. Neural cysts form one of the subgroups, in which is included colobomatous cyst associated with ocular maldevelopment, congenital cystic eye, and cysts associated with brain and meningeal tissue (cephalocele and optic nerve meningocele).[5]

The presentation of microphthalmos with a cyst can be as a protruding mass in the inferior orbit associated with a malformed microphthalmic eye; the cyst may be so small that it cannot be detected clinically or it may be so large that it obscures the globe.[6] These eyes usually have a poor visual outcome. Foxman and Cameron[7] reported that bilateral microphthalmos with colobomatous cyst may be associated with major systemic abnormalities (central nervous system, renal, or cardiovascular), whereas unilateral involvement is usually associated with minor abnormalities. Our case presented with thinning of the corpus callosum and mild mental retardation, besides cleft lip and palate.

Imaging is of primary importance in the evaluation of cystic lesions of the orbit. It helps to differentiate colobomatous cyst from optic nerve sheath cysts, cephalocele, and optic nerve meningocele as well as from solid tumors of the orbit.[8] Imaging also helps in revealing any communication between the cyst and the globe. This information is useful when planning the management of these lesions.

Management of these cysts depends on the age of the patient, the size of the cyst, the presence of communication between the globe and the cyst, and the visual prognosis.[9] Surgical management varies from simple aspiration of the cyst, enucleation of the microphthalmic eye along with the cyst, and excision of the cyst with preservation of the globe. We managed our case with cyst excision. This was the best approach in this case as the child had normal bony growth. Cystic lesions of the orbit are rare malformations. Imaging plays a vital role in differentiating these lesions from other tumors. For a relatively good cosmetic outcome, these cases are best managed by globe-salvaging surgery.
